# A Special Extract of *Bacopa monnieri* (CDRI-08)-Restored Memory in CoCl_2_-Hypoxia Mimetic Mice Is Associated with Upregulation of *Fmr-1* Gene Expression in Hippocampus

**DOI:** 10.1155/2015/347978

**Published:** 2015-08-27

**Authors:** Anupama Rani, S. Prasad

**Affiliations:** Biochemistry & Molecular Biology Lab, Centre of Advanced Study in Zoology, Banaras Hindu University, Uttar Pradesh, Varanasi 221005, India

## Abstract

Fragile X mental retardation protein (FMRP) is a neuronal translational repressor and has been implicated in learning, memory, and cognition. However, the role of *Bacopa monnieri* extract (CDRI-08) in enhancing cognitive abilities in hypoxia-induced memory impairment via *Fmr-1* gene expression is not known. Here, we have studied effects of CDRI-08 on the expression of *Fmr-1* gene in the hippocampus of well validated cobalt chloride (CoCl_2_)-induced hypoxia mimetic mice and analyzed the data with alterations in spatial memory. Results obtained from Morris water maze test suggest that CoCl_2_ treatment causes severe loss of spatial memory and CDRI-08 is capable of reversing it towards that in the normal control mice. Our semiquantitative RT-PCR, Western blot, and immunofluorescence microscopic data reveal that CoCl_2_-induced hypoxia significantly upregulates the expression of Hif-1*α* and downregulates the *Fmr-1* expression in the hippocampus, respectively. Further, CDRI-08 administration reverses the memory loss and this is correlated with significant downregulation of Hif-1*α* and upregulation of *Fmr-1* expression. Our data are novel and may provide mechanisms of hypoxia-induced impairments in the spatial memory and action of CDRI-08 in the recovery of hypoxia led memory impairment involving *Fmr-1* gene encoded protein called FMRP.

## 1. Introduction

Brain requires a continuous supply of oxygen to perform its normal function. Being the largest consumer of oxygen, it is especially sensitive to hypoxia, a condition in which brain receives reduced oxygen. Several studies have shown that injury to the brain due to loss of oxygen triggers memory loss and causes learning and memory deficits [1, 2]. Although the whole brain is susceptible to hypoxia, hippocampus in particular has been reported to be severely affected by hypoxia [3, 4] as it plays crucial roles in encoding and consolidating memory [5, 6].

Memory formation, maintenance, and retrieval are dynamic processes involving transcription, translation, and expression of several proteins [7]. Fragile X mental retardation protein (FMRP), an mRNA-binding protein [8–11], is prevalently present in dendritic spines [12] and regulates protein synthesis relevant to synaptic plasticity [10]. FMRP was first characterized in context of the fragile X syndrome (FXS) which results from loss of function mutations in* Fmr-1* gene, which in turn results in mental retardation, loss of memory, and abnormal cognitive behavior in fragile X mental retardation syndromes (FXS). FMRP is a 70–80 KDa protein abundantly expressed in brain and testis [13, 14]. FMRP-mediated translational regulation plays important roles in proper synaptic connectivity [15] and plasticity [16, 17]. Since the dendritic protein synthesis required for achieving synaptic plasticity is under the strict control [18, 19], any change in the level of FMRP may lead to alterations in the synaptic plasticity, thus learning and memory. Whether hypoxia leads to any alterations in the expression of* Fmr-1* gene is not known. Therefore, we have examined the effects of hypoxia on the expression of the* Fmr-1* gene at transcript and protein levels in relation to learning and memory in cobalt chloride-induced hypoxia mimetic mouse model.

*Bacopa monnieri,* also known as Brahmi, is a traditional Ayurvedic medicinal plant and it has been extensively used in India as a nerve tonic for centuries [20]. In the Indian Ayurvedic system of medicine,* Bacopa monnieri *belongs to a group of medicine called “Medhya Rasayana” which is known to act on nervous system and improve mental abilities by enhancing memory and tunes cognition.* Bacopa monnieri *extract contains mixture of saponins, for example, bacoside A, bacopasides I and II, bacopasaponin C, and flavonoids [21–23], as active constituents. Its extract has been reported to facilitate cognitive functions as well as to augment mental retention capacity. There is evidence that the mechanism of action of* Bacopa monnieri *could be attributed to a combination of cholinergic modulation [24–27] and antioxidant effects [28–31]. Although many reports suggests the nootropic capabilities of* Bacopa monnieri *extract, its effect on* Fmr-1 *gene expression in relation to learning and memory has not been studied to date. In the light of the crucial role played by the* Fmr-1 *gene encoded FMRP in the formation and maintenance of synaptic connectivity, it is possible that* Fmr-1 *gene could be one of the targets of bacoside's action during memory enhancement. Therefore, in the present study, we have investigated whether hypoxic condition leads to any alteration in spatial memory and this alteration is associated with change in the expression of FMRP in the hippocampus of cobalt chloride-induced hypoxic mouse model [32], and further we studied whether a selected dose of CDRI-08 (obtained from a pilot study) recovers the alteration in spatial memory and reverses alterations in the* Fmr-1* gene expression in the hippocampus due to hypoxia.

## 2. Materials and Methods

### 2.1. Materials

A standardized extract of* Bacopa monnieri* (CDRI-08) containing 58.18% of bacosides was received as a kind gift from Dr. H. K. Singh, Director, Lumen Research Foundation, Chennai, India. Cobalt chloride was purchased from Sisco research laboratory, India (SRL). All other chemicals and reagents were of analytical grade and purchased from Merck, India, and Sigma Aldrich, USA.

### 2.2. Animals and Drug Treatment

Male Swiss albino mice of age 20 ± 5 weeks, weighing 25 ± 5 g were used in the present study. Mice were housed in the animal house maintained at 25 ± 2°C with alternating 12 h light and dark cycles, access to standard mice feed and water* ad libitum*. All experimental procedures were approved by the ethical committee of Banaras Hindu University. Prior to exposure to hypoxia mimetic condition mice were trained in Morris water maze for 8 days. After training, mice were randomly divided into six groups (*n* = 7 mice per group) for different treatments as (1) control group (C) administered with 5% Tween 80, (2) Brahmi group orally treated with standardized dose of* Bacopa monnieri *extract (CDRI-08) (200 mg/Kg BW) dissolved in 5% Tween-80 for 8 days, (3) hypoxia group 1 (HA) in which mice were administered with standardized dose of cobalt chloride (40 mg/kg BW) for 15 days [32] and then were kept for 8 days without any treatment to check if hypoxic condition reverts back to normal in this time period, (4) hypoxia group 2 (HB) in which mice were administered with standardized dose of cobalt chloride (40 mg/kg BW) for 15 days, (5) mice who were orally administered with CDRI-08 (200 mg/Kg BW in 5% Tween-80) for 8 days as mentioned above followed by induction of hypoxic condition (B + H), (6) and mice were first orally treated with cobalt chloride followed by treatment with CDRI-08 (200 mg/Kg BW in 5% Tween-80) for 8 days (H + B). After completion of respective treatments, mice of all the groups were subjected to Morris water maze test. The animals were sacrificed and the brain was dissected out on ice. The hippocampus was removed for RNA isolation and protein lysate preparation for gene expression studies. For preparation of cryostat brain sections, the mice were anesthetized using 50 mg/Kg BW sodium pentobarbital and perfused with 4% paraformaldehyde in PBS before sacrificing.

### 2.3. Morris Water Maze Test

Morris water maze test, a well established behavioral test for evaluation of spatial navigation memory in rodents, was performed on the experimental mice following the procedure of Morris et al. [5]. The Morris water maze consisted of a black circular tank (106 cm diameter, 76.2 cm height) filled with water up to 1/3 height maintained at a temperature of 24 ± 2°C. A Plexiglas escape platform (9.5 cm × 35 cm) was submerged at a fixed position 1 cm below the water surface. Distinct geometric visual cues were fixed in each quadrant at specific locations which were visible to mice while under training and test. Performance of mice in the maze was recorded by video camera suspended above the maze and interfaced with a video tracking system (ANY-maze software, Microsoft version 4.84, USA). Mice were given an acclimatization session of 60 s in the water maze 2 days before the start of training. The training consisted of 3 trials each of 90 s/day with an intertrial interval of 5 min for 8 days. Each trial consisted of gently placing the mice by hand into the water, facing the wall of the pool and being allowed to swim freely for 90 s and find the hidden platform. Mice which failed to locate the platform within 90 s were guided to the platform and allowed to remain on the platform for 15 s. After the completion of training period of 8 days, mice were divided randomly into six groups as described earlier and after the completion of all treatments, Morris water maze test was performed to investigate hypoxia induced loss of memory and evaluation of its recovery by CDRI-08. Alteration in spatial learning and memory was assessed in terms of latency (sec) and path length (m). Latency is defined as the time taken by mice to locate the hidden platform, expressed in sec whereas the path length is defined as distance travelled by mice to reach the hidden platform, expressed in m. In the probe-trial experiment in which the hidden platform was removed alteration in memory was studied in terms of time spent in target quadrants and number of platform crossings to infer the strength of the memory of the mouse for locating the platform. 

### 2.4. Cryosectioning and Immunofluorescence Detection of FMRP

To study the* in situ *expression of FMRP, first 15–20 mL of normal saline was passed transcardially to flush out the blood. Thereafter, intra-arterial perfusion of 4% paraformaldehyde solution was given. The brain was dissected out and was kept in paraformaldehyde medium at 4°C overnight. Then the brain tissues were cryopreserved in different grades of sucrose, that is, 10%, 20%, and 30% sucrose. Finally, cryosectioning was carried out using HM525 Microcryotome and sections of 15 *μ*m thickness were obtained. Cryosections were washed in PBS and were then permeabilized by soaking in 0.3% triton X-100 in PBS medium for 10 min, washed in 1X PBS for 5 min, and were kept in blocking solution containing 5% goat serum, 0.2% Tween-20, and 0.2% NP-40 in PBS for 3 hrs at RT. Thereafter, the sections were incubated in anti-FMRP primary antibody (1 : 200 dilution; Sigma Aldrich), overnight at 4°C followed by washing in 1X PBS for 15 min. The sections were then incubated with FITC conjugated goat anti-rabbit IgG (1 : 500 dilution; Bangalore Genei) for 4 hrs at RT in dark. Sections were then mounted in fluoroshield mounting medium containing DAPI and photographs were taken at 540 nm for FITC and 460 nm for DAPI at 20x magnification using Nikon 90i Motorized Research Microscopy, equipped with NIS Elements 4.0 AR software. The immunofluorescence intensity was analyzed as integrated densitometric value (IDV) using Image J software.

### 2.5. Total RNA Isolation

Total RNA from the hippocampal samples was isolated using TRI reagent (Sigma, USA) following the suppliers manual. The aqueous phase was collected and mixed with equal volume (v/v) of isopropanol and precipitated at –70°C. The RNA pellet was collected, washed with ice-chilled 70% ethanol, and dissolved in DEPC-treated water. Extracted RNA was treated with DNase-I (DNAfree, Ambion) according to the manufacturer's guidelines to remove any DNA contamination. RNA content was determined by measuring the absorbance at 260 nm using UV-Visible Spectrophotometer and its integrity was checked by 1% formaldehyde agarose gel electrophoresis following the procedure described earlier [33], and quality of its preparation was found suitable for RT-PCR experiment (results not shown).

### 2.6. Semiquantitative RT-PCR

To carry out semiquantitative RT-PCR, cDNA strands were synthesized in each case by mixing 2 *μ*g of the DNA free total RNA and 200 ng random hexamer primers (MBI Fermentas, USA) in 11 *μ*L reaction volume and incubating the whole mix at 70°C for 5 min. Thereafter, 2 *μ*L of 5X reaction buffer, 2 *μ*L of 10 mM dNTP mix, and 20U of RNase inhibitor (Ribolock, MBI Fermentas, USA) were added, and the volume was made up to 19 *μ*L. The tube was incubated for 5 min at 25°C, and 200U of M-MuLv reverse transcriptase (New England Biolabs) was added. Further, the tube was incubated for 10 min at 25°C initially and then at 42°C for 1 h in the thermal cycler (G-Storm, UK). The reaction was terminated by heating the reaction mix at 70°C for 10 min followed by its incubation at 4°C.

The resulting cDNA was used as template to carry out polymerase chain reaction using thermal cycler (G-Strom, UK). PCR reactions were carried out in a 25 *μ*L reaction mixture containing 2 *μ*L cDNA, 1X Taq polymerase buffer with MgCl_2_, 0.2 mM of each dNTP (MBI Fermentas, USA), 1.0 unit of Taq DNA polymerase (Banglore Genei, India), and 10 pmol of appropriate primers (as shown in Table 1). Reactions were carried out using thermal cycler (G-Strom, UK) with the reaction conditions as described in Table 1. The amplified products were resolved by 2% agarose gel electrophoresis and detected by ethidium bromide staining. The ethidium bromide stained gels were photographed and intensity of the bands as described above was scanned and quantified using Alpha Imager 2200 software separately to obtain integrated density values (IDV) and were normalized with that of *β*-actin to obtain the relative density values (RDV) for individual amplicons.

### 2.7. Total and Nuclear Lysate Preparation

For western blot analysis, the cytosolic and nuclear proteins lysate were prepared following the procedure as described earlier [32]. Briefly, the protein lysate from hippocampus was prepared in the buffer containing 20 mmol/L HEPES, 10 mmol/L KCl, 1 mmol/L EDTA, 1 mmol/L dithiothreitol, 0.2% NP40, 10% glycerol, 1 mmol/L PMSF, and 1 *μ*g/mL protease inhibitor cocktail. After 5 minutes of incubation on ice, the samples were centrifuged at 13,000 ×g for 10 minutes. The supernatants (cytosolic extracts) thereafter were used for the western blot analysis of FMRP. The resulting pellets were suspended in 50 *μ*L buffer containing 350 mmol/L NaCl, 20% glycerol, 20 mmol/L HEPES, 10 mmol/L KCl, 1 mmol/L EDTA, 1 mmol/L PMSF, 20% SDS, 10% Sodium deoxycholate, and 1 *μ*g/mL protease inhibitors cocktail and the suspension was vigorously mixed with finger tips and incubated on ice for 30 minutes. Thereafter, samples were centrifuged at 13,000 ×g for 10 minutes at 4°C and the resulting supernatants (nuclear extracts) were used for detection of hypoxia marker protein HIF-1*α*. The total protein contents in both the preparations were estimated by Bradford method using bovine serum albumin as standard [34].

### 2.8. Western Blot Analysis

In order to examine the levels of expression of HIF-1*α* and FMRP a uniform 50–80 *μ*g of the protein lysate were resolved by SDS-polyacrylamide gel electrophoresis [35] and transferred onto PVDF membrane by wet transfer method. The membrane was blocked with 5% nonfat milk in PBS (35 mM NaCl, 8 mM Na_2_HPO_4_, 5 mM KCl, 7 mM KH_2_PO_4_, pH 7.4) medium for 4 h at room temperature. The blot was then incubated using rabbit polyclonal antibodies for HIF-1*α* (1 : 1000; Cayman, USA) and FMRP (1 : 2500; Sigma Aldrich) in 5% nonfat milk in PBS (pH 7.4) overnight at 4°C. The blot was further treated with secondary antibody against mouse IgG conjugated with horse radish peroxidase (1 : 2,500) in blocking buffer for 6 h at room temperature. Horse radish peroxidase (HRP) conjugated antibody for *β*-actin (1 : 25,000; Sigma) was used for the detection of *β*-actin as internal control. HIF1*α*, FMRP and *β*-actin (internal control) signals were detected by enhanced chemiluminescence (ECL) method and the intensity of resulting signals on the X-ray film were scanned and quantified using Alpha Imager 2200 software separately. Scan data of proteins as mentioned above was normalized with that of the *β*-actin to obtain relative densitometric value (RDV).

### 2.9. Statistical Analysis

All the experiments were repeated thrice. Data were expressed as mean ± standard error means (S.E.M.). Results obtained from Morris water maze test were analyzed by One way ANOVA followed by post hoc least significance difference test (LSD). For analysis of the molecular data, Tukey's post hoc test was used after one way ANOVA. *P* value < 0.05 was considered statistically significant.

## 3. Results

### 3.1. CDRI-08 Attenuates Hypoxia Induced Alteration in the Spatial Learning and Memory

**Figure 1:**
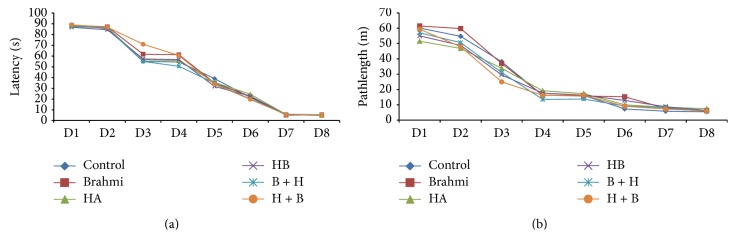
Latency of mice of various groups during training in Morris water maze (a). Path length of mice during training in Morris water maze (b).

**Figure 2:**
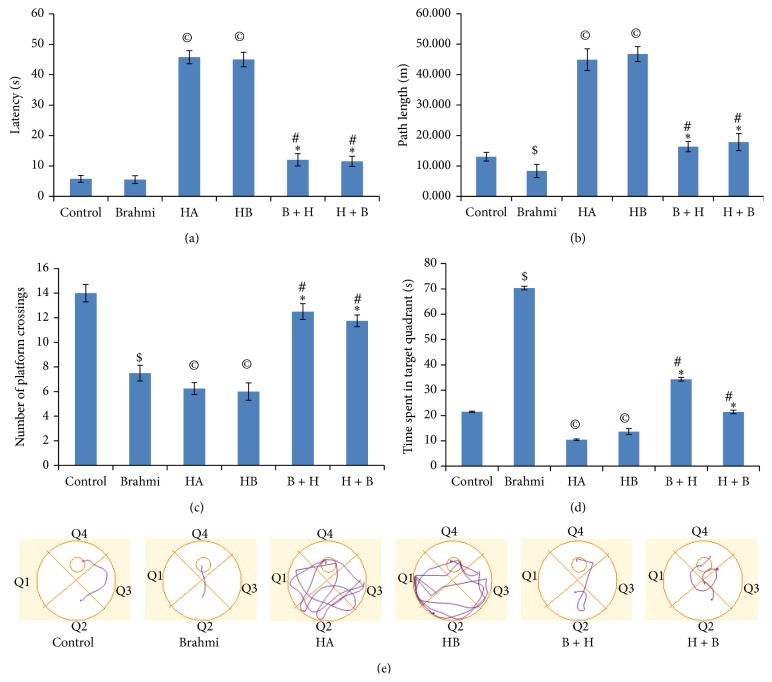
Effects of CDRI-08 on spatial memory of hypoxic mice. (a) Pattern of latency; (b) path length; (c) number of platform crossing, and (d) time spent in target quadrant. Values are expressed as mean ± S.E.M.  ^$^ *P* < 0.05 versus control; ^©^ *P* < 0.05 versus control;  ^∗^ *P* < 0.05 versus HA; ^#^ *P* < 0.05 versus HB. (e) Track record for probe trail of mice following exposure to hypoxia mimetic condition and CDRI-08 treatment. Q1: Quadrant 1; Q2: Quadrant 2; Q3: Quadrant 3; Q4: Quadrant 4 (target quadrant). Mice administered with BME: Brahmi; HA, treatment of CoCl_2_ for 15 days to induce hypoxia; HB: hypoxic mice left for 8 days without any treatment after CoCl_2_ treatment for 15 days; B + H: mice pretreated with CDRI-08 followed by CoCl_2_ treatment; H + B: CDRI-08 treated hypoxic mice.

As shown in Figure 1(a), training of mice for 8 days in Morris water maze leads to progressive improvement of acquisition, the ability of mice to explore the hidden platform in the target quadrant. The decline in latency time indicates that mice got trained with task given on the maze. This is further evident by decrease in path length (Figure 1(b)). Exposure to hypoxic condition resulted in significant increase (*P* < 0.05) in the latency and path length as compared to the control group. The above finding can well be seen in Figures 2(a), 2(b), and 2(c). Hypoxic conditions brought about by cobalt chloride treatment for 15 days (HA) and withdrawal of the treatment for next 8 days (HB) show similar effects. During these conditions, the hypoxia significantly decreased the acquisition of information and poor consolidation as evident by significant increase in the latency period and the path length. The control mice administered with CDRI-08 showed significant decrease in the latency time as well as path length as compared to control mice. Both pre- and posthypoxic treatment of mice with the CDRI-08 caused significant decline (*P* < 0.05) in latency (Figure 2(a)) and path length (Figure 2(b)) as compared to hypoxic groups. In the probe trial test, in which the hidden platform was removed, mice in the hypoxic conditions (HA and HB) showed significant decrease (*P* < 0.05) in number of platform crossings (Figure 2(c)) and time spent in the target quadrant (Figure 2(d)) as compared to the control group. Conversely, the hypoxic mice which were given pre- and posttreatment of CDEI-08 showed a significant increase (*P* < 0.05) in the number of platform crossings (Figure 2(c)) and time spent in the target quadrant (Figure 2(d)) as compared to hypoxic groups (HA and HB). Also, we observe that the CDRI-08, when administered to normal control mice, the number of platform crossing is significantly reduced and the time spent in the target quadrant is significantly increased. This indicates that the CDRI-08 possesses the ability of enhancing spatial learning and memory.

### 3.2. CDRI-08 Ameliorates Hypoxia Induced Expression of HIF-1*α* in the Hippocampus

**Figure 3:**
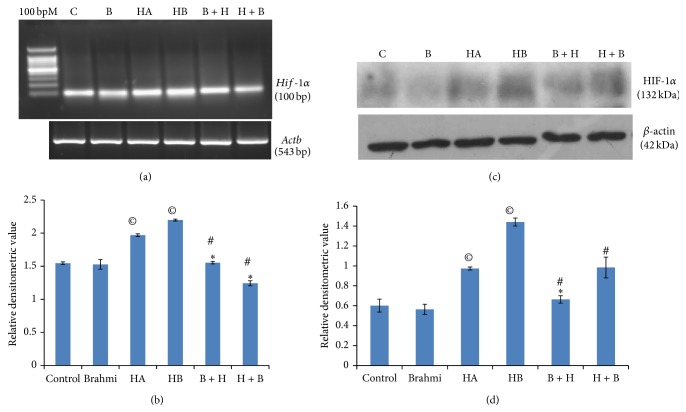
Effects of hypoxia and CDRI-08 on* Hif-1α* gene expression. Semiquantitative RT-PCR analysis of* Hif-1α* (a) and Western blot analysis of HIF-1*α* (c). Bar shows the relative density value developed by integrated densitometric values (IDV) of HIF-1*α* by IDV of *β*-actin. Each bar represents the mean ± SEM. ^©^ *P* < 0.05 versus control;  ^∗^ *P* < 0.05 versus HA; ^#^, indicates *P* < 0.05 versus HB.

Our RT-PCR analysis data indicate that expression of* Hif-1α* mRNA is significantly upregulated (*P* < 0.05) due to hypoxia in both the conditions (HA and HB) as compared to control group (Figure 3(a)). CDRI-08 when administered to mice before hypoxia was generated (prehypoxic treatment) and after the hypoxia (posthypoxi treatment) as described above, significantly downregulated the level of* HIF-1α* mRNA (*P* < 0.05) towards the normal as compared to both hypoxic conditions. Our Western blot data reveals that the level of HIF-1*α* protein is significantly upregulated (*P* < 0.05) in the hippocampus of hypoxic mice of both HA and HB conditions; however, its level is prominently higher in HB conditions as compared to normal control, which confirms the establishment of hypoxic condition (Figure 3(b)). CDRI-08 treatment to hypoxic mice (prehypoxic and posthypoxic) was found to significantly downregulate the level of HIF-1*α* protein (*P* < 0.05) towards that in the normal control mice.

### 3.3. Effect of Hypoxia and* Bacopa monnieri* Extract on* Fmr-1* mRNA Expression in the Hippocampus

**Figure 4:**
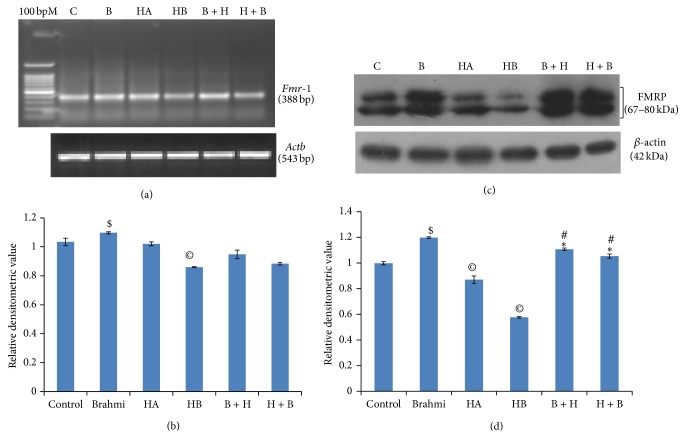
Effects of hypoxia and CDRI-08 on* Fmr-1* gene expression. Semiquantitative RT-PCR analysis of* Fmr-1* (a) and Western blot analysis of FMRP (c). Bar shows the relative density value developed by integrated densitometric values (IDV) of HIF-1*α* by IDV of *β*-actin. Each bar represents the mean ± SEM.  ^©^ *P* < 0.05 versus Control;  ^∗^ *P* < 0.05 versus HA; ^#^, indicates *P* < 0.05 versus HB.

As shown in Figures 4(a) and 4(b), our RT-PCR data shows that hypoxia does not affect the level of* Fmr-1* mRNA in the hippocampus in the initial phase of hypoxia (HA); however, its level is significantly downregulated during the period when hypoxia continued without cobalt chloride treatment (HB) as compared to normal control (*P* < 0.05). Pretreatment with CDRI-08 or posthypoxic CDRI-08 treatment did not show any significant change in the level of* Fmr-1* transcript.

### 3.4. CDRI-08 Causes Recovery of FMRP Expression in the Hippocampus of Hypoxic Mice

**Figure 5:**
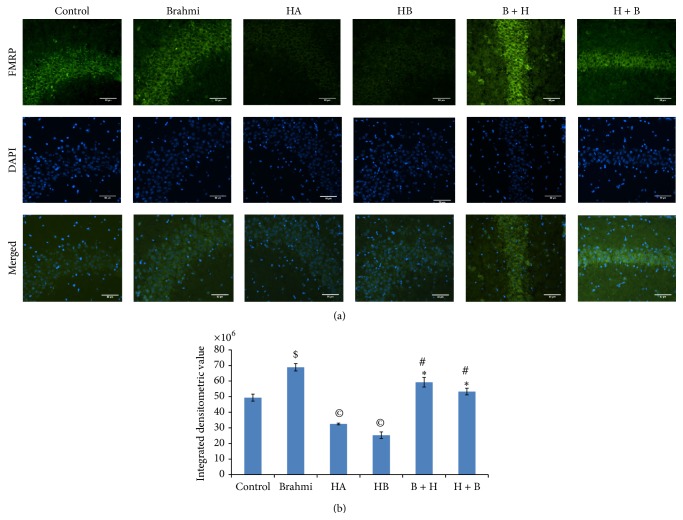
Effects of hypoxia and CDRI-08 on the expression of FMRP in hippocampus. Photomicrophotographs shows immunofluorescence (20x magnification) illustrating FITC-labeled signals of FMRP in CA3 region of hippocampus (a). Data were calculated in terms of integrated densitometric value (b). Bar represents the mean ± SEM.  ^$^ *P* < 0.05 versus control,  ^©^ *P* < 0.05 versus control,  ^∗^ *P* < 0.05 versus HA, ^#^ *P* < 0.05 versus HB, Scale bar = 1 *μ*.

**Figure 6:**
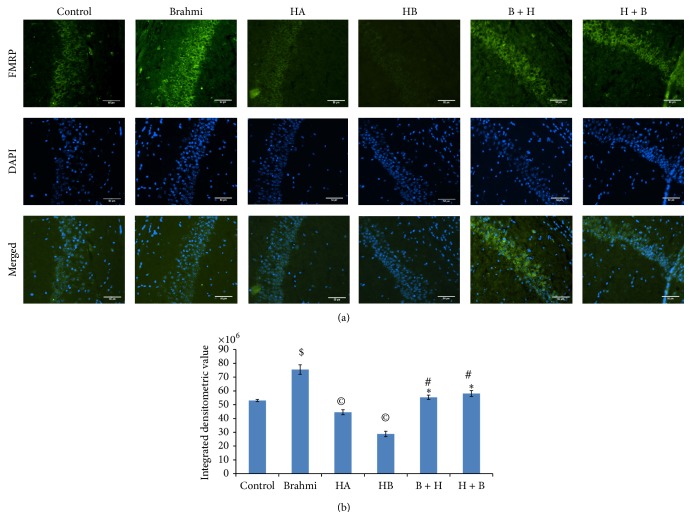
Effect of hypoxia and CDRI-08 on the expression of FMRP in hippocampus. Photomicrophotographs shows immunofluorescence (20x magnification) illustrating FITC-labeled signals of FMRP in CA1 region of hippocampus (a). Data were calculated in terms of integrated densitometric value (b). Bar represents the mean ± SEM.  ^$^indicates *P* < 0.05 versus Control, ^©^ *P* < 0.05 versus Control,  ^∗^ *P* < 0.05 versus HA, ^#^ *P* < 0.05 versus HB, Scale bar = 1 *μ*.

Western blot data reveal that hypoxic conditions (HA and HB) cause significant downregulation of the FMRP level; however, the decline was more prominent in the withdrawal period (HB) (*P* < 0.05). On the other hand, administration of CDRI-08 to the normal control mice caused significant upregulation of FMRP expression (*P* < 0.05) as compared to that in the control. Treatment of mice with CDRI-08 before hypoxic condition (B + H) and after hypoxic condition (H + B) both causes significant upregulation in the level of FMRP when compared to hypoxic condition. Mice* pretreated with CDRI-08 followed by CoCl*_*2*_ *and the CoCl*_*2*_ *-(hypoxic) mice treated with CDRI-08 resulted in significant upregulation in the expression of FMRP in the hippocampus as compared to mice of hypoxic groups (HA and HB)* (Figures 4(c) and 4(d)). These results were further confirmed by immunofluorescence microscopic based studies on the* in situ* detection of FMRP expression in brain sections in CA3 (Figures 5(a) and 5(b)) and CA1 (Figures 6(a) and 6(b)) regions of the hippocampus showed the patterns similar to that in Western blot results.

## 4. Discussion

Use of herbal preparations in the treatment of nervous disorders and many other diseases has tremendously increased especially in the last decade. These preparations are rich in multiple active components and have emerged as preferred prophylactic agents owing to their wide spectrum therapeutic benefits and minimum risks due to significantly less side effects as compared to their synthetic variants.* Bacopa monnieri* is one of the plants that have been widely used in Indian medicinal system of Ayurveda for the treatment of various nervous disorders in general and memory related diseases in particular [36]. In the present study, we have used alcoholic extract of* Bacopa monnieri* named CDRI-08 which is well characterized to be rich in Bacoside A and Bacoside B and studied its effects on the cobalt chloride-induced hypoxia led loss of spatial learning and memory and its effects on the expression of fragile X mental retardation protein (FMRP), one of the proteins that regulate synaptic plasticity, a neurophysiological mechanism underlying learning, memory, and cognition. In order to assert the learning and memory loss in mice due to hypoxia and the possible action of CDRI-08 in restoring the learning and memory loss, we chose to use the Morris water maze paradigm as this test has been often used to assess the alterations in hippocampal spatial learning and memory in rodents [37]. 

Our data suggests that hypoxia, during which the normal oxygen supply is reduced to organs including brain, causes impairments in the learning and memory consolidation process. Also, it reveals that when hypoxic condition is prolonged further without any further treatment of cobalt chloride (hypoxia withdrawal effects), the cognitive impairment effects are similar to hypoxic conditions with continuous cobalt chloride treatment for the experimental period. This indicated that the period of withdrawal had no separate effects on the level of memory impairment (Figures 1 and 2). This impairment in the spatial memory due to hypoxia could be attributed to rise in the level of the hypoxia marker protein Hif-1*α*, a transcription factor which regulates the early hypoxia responsive genes including glutamate transporter type-1 (GLUT-1), erythropoietin (Ep), and late responsive genes like superoxide dismutase (SOD) and catalase (CAT) and many proteins related to synaptic plasticity [38]. In order to confirm whether Hif-1*α* expression is altered and associated with decline in learning and memory, we examined alteration in its expression in the hippocampus of the normal control and experimental mice. It was observed that the hypoxia-induced memory impairment in mice is related with enhanced expression of Hif-1*α*, which could have affected the levels of the antioxidative stress enzymes such as SOD and CAT. This data corroborates with our earlier findings on the relation between increased Hif-1*α* level due to hypoxia and decline in the activities and expression of these enzymes [38]. Our data suggest that effects of hypoxia might not differ much once that hypoxia led neurological derangement has occurred. Memory impairment due to hypoxia, as evident from rise in the level of Hif-1*α*, may be due to possible alterations in expression of synaptic plasticity related proteins such as AMPA, NMDA, and metabotropic glutamate receptors (AMPAR, NMDAR, and mGluR) which control long term potentiation (LTP) or long term depression (LTD), the cellular basis of learning and memory [39–42]. Our data demonstrated that cobalt chloride induced hypoxic condition resulted in alteration in spatial memory which is found to be in accordance with several other studies which report that chronic exposure to hypobaric hypoxia leads to memory impairment in rats [43, 44]. We also observed that CDRI-08 treatment to hypoxic mice improves their impaired spatial memory which can be understood by significant decrease in the latency and path length along with significant increase in number of platform crossings and time spent in the target platform. This can be correlated with the neuroprotective role of the CDRI-08 in restoration of the altered spatial memory towards the normal condition. Similar role of CDRI-08 has been shown in earlier studies where CDRI-08 plays positive roles in animals affected with altered spatial memory due to hypobaric hypoxia [45], Alzheimer's disease [46], and scopolamine-induced amnesia [47].

As HIF-1*α* is hallmark of hypoxic condition, it is possible that CDRI-08 alters its expression or its stability. Our results, to our surprise, show upregulation of* Hif-1α* at both transcript and protein levels in the hippocampus of CoCl_2_-induced hypoxic mice and it was found that CDRI-08 treatment to hypoxic mice reversed the level of Hif-1*α* towards that in the normal mice. Therefore, it is suggestive that CDRI-08 treatment based restoration of learning and memory is correlated with the levels of HIF-1*α*. Also, from our study, it can be concluded that CDRI-08 has similar effects whether it is given before hypoxia is developed or after the hypoxia was developed. However, the precise mechanism by which CDRI-08 modulates the expression of HIF-1*α* and which thereby protects or restores memory cannot be assertively explained by our results and it is needed to be thoroughly studied. Nonetheless, CDRI-08's positive role in impaired spatial learning and memory is evident from our studies. The CDRI-08 treatment-dependent restoration of memory in hypoxia caused decline in learning and memory might be attributed to its free radical scavenging function [28–31] and cholinergic modulation [24–27] which are being investigated in our group.

As indicated earlier in the discussion, cobalt chloride-dependent hypoxic condition that decreases the level of learning and memory may also be correlated with alterations in the synaptic plasticity related proteins. Since FMRP is one of the proteins that regulate LTP and LTD via regulation of various glutamate receptors like AMPAR, NMDAR, and mGluR, it is likely that hypoxia may cause decline in memory, and CDRI-08 treatment reverses the impaired memory towards that in the normal control, which may be due to alterations in the level of FMRP which might in turn affect synaptic plasticity. Therefore, we thought to examine alterations in the expression of* Fmr-1* gene at transcript as well as protein levels. We observed that impairment in spatial memory was significantly correlated with the expression of FMRP, an important protein associated with synaptic plasticity. We report here for the first time that hypoxic condition leads to a remarkable decrease in hippocampal* Fmr-1* expression at both mRNA and protein levels as analyzed by RT-PCR and Western blotting. Consistent with the Western blotting results, the immunofluorescence studies also revealed remarkable downregulation of FMRP in the hippocampus of mice of both the groups of hypoxic mice, and it upregulates its expression in the CDRI-08-treated prehypoxic or hypoxic mice towards its level in the normal control mice. Our results are consistent with the findings which show impairment in spatial memory in* Fmr-1* knockout mice [48, 49] suggesting a crucial role of FMRP in the hypoxia led memory impairments and CDRI-08-dependent memory restoration processes. The expression of FMRP is reported be high in the hippocampus [50] and since, the hippocampus has been shown to be necessary for memory in humans and rodents, specifically for the formation of spatial memory in rodents, FMRP seems to play a critical role in the function of hippocampus. FMRP is found in dendritic spines [51], the important postsynaptic sites of plasticity induction and maintenance, it plays role in the regulation of dendritic mRNA translation [11, 52] which is required for multiple forms of plasticity [53] and it is dynamically regulated by activity-dependent synaptic activation can trigger its local translation and rapid degradation [54], it is established that FMRP is a candidate protein involved in regulating synaptic plasticity. Other studies have revealed that translation of proteins regulated by FMRP includes microtubule-associated protein 1B (MAP1B) and activity-regulated cytoskeleton-associated protein (ARC) [55, 56]. Studies have shown that* Fmr-1* promoter possesses the CRE site that binds CREB in the regulation of its own transcription in neural cells [57, 58]. In a recent study, it has been shown that CREB may specifically contribute to the upregulation of FMRP by stimulating Group I mGluRs [59], suggesting the CREB-dependent regulation of FMRP level. Therefore, it can be speculated that the hypoxia-induced decline in the learning and memory may be due to alterations in above to which FMRP is intricately associated which in turn might cause defects in synaptic plasticity. A recent report suggests that the chronic administration of* B. monniera* extract improves cognitive behavior by upregulation of PKA, MAPK and pCREB. Our study also reveals that CDRI-08 upregulates FMRP expression and it is likely to possess the neuroprotective or restorative effects, respectively, by way of FMRP-dependent regulation of pCREB and its binding with CRE site on the* Fmr-1* gene promoter leading to transcriptional regulation of* Fmr-1* and several other genes which in turn may facilitate the role of synaptic proteins and synaptic plasticity, hence learning and memory.

Although various reports on Bacosides have suggested its antioxidant properties [60] and cholinergic property [26] which contributes in restoration of altered memory by* Bacopa monnieri*, based on the strength of available publications, we can claim that our report is novel on the effects of* Bacopa monnieri* on the expression of* Fmr-1* gene and its association with spatial memory. Thus our study suggests a possible mechanism for the hypoxia-induced memory loss involving FMRP and the mode of action of CDRI-08 during recovery of memory impaired due to hypoxia, which needs to be addressed in more details.

## Figures and Tables

**Table 1:** Details of gene specific primer sequences, temperature conditions, cycle numbers and amplicon sizes.

Genes	Primers	PCR condition		Amplicon size
*Hif-1a *	F 5′-AGACAGACAAAGCTCATCCAAGG-3′ R 5′GCGAAGCTATTGTCTTTGGGTTTAA-3′	94°C - 3′		100 bp
		94°C - 45′′	30 cycles	
		59°C - 30′′		
		72°C - 45′′		

*Fmr-1 *	F 5′-TTACAGAAATAGGGGGCACG-3′ R 5′-TACGCTGTCTGGCTTTTCCT-3′	94°C - 3′		388 bp
		94°C - 45′′	34 cycles	
		59°C - 30′′		
		72°C - 45′′		

*Actb *	F 5′-ATCGTGGGCCGCTCTAGGCACC-3′ R 5′CTCTTTGATGTCACGCACGATTTC-3′	94°C - 3′		543 bp
		94°C - 45′′	28 cycles	
		57°C - 30′′		
		72°C - 45′′		
